# Geraniol Averts Methotrexate-Induced Acute Kidney Injury via Keap1/Nrf2/HO-1 and MAPK/NF-κB Pathways

**DOI:** 10.3390/cimb43030123

**Published:** 2021-10-24

**Authors:** Nancy S. Younis, Heba S. Elsewedy, Tamer M. Shehata, Maged E. Mohamed

**Affiliations:** 1 Department of Pharmaceutical Sciences, College of Clinical Pharmacy, King Faisal University, Al-Ahsa 31982, Saudi Arabia; helsewedy@kfu.edu.sa (H.S.E.); tshehata@kfu.edu.sa (T.M.S.); memohamed@kfu.edu.sa (M.E.M.); 2 Department of Pharmacognosy, College of Pharmacy, Zagazig University, Zagazig 44519, Egypt

**Keywords:** apoptosis, essential oil, inflammatory mediators, monoterpene, renal injury

## Abstract

Objectives: Geraniol, a natural monoterpene, is an essential oil component of many plants. Methotrexate is an anti-metabolite drug, used for cancer and autoimmune conditions; however, clinical uses of methotrexate are limited by its concomitant renal injury. This study investigated the efficacy of geraniol to prevent methotrexate-induced acute kidney injury and via scrutinizing the Keap1/Nrf2/HO-1, P38MAPK/NF-κB and Bax/Bcl2/caspase-3 and -9 pathways. Methods: Male Wister rats were allocated into five groups: control, geraniol (orally), methotrexate (IP), methotrexate and geraniol (100 and 200 mg/kg). Results: Geraniol effectively reduced the serum levels of creatinine, urea and Kim-1 with an increase in the serum level of albumin when compared to the methotrexate-treated group. Geraniol reduced Keap1, escalated Nrf2 and HO-1, enhanced the antioxidant parameters GSH, SOD, CAT and GSHPx and reduced MDA and NO. Geraniol decreased renal P38 MAPK and NF-κB and ameliorated the inflammatory mediators TNF-α, IL-1β, IL-6 and IL-10. Geraniol negatively regulated the apoptotic mediators Bax and caspase-3 and -9 and increased Bcl2. All the biochemical findings were supported by the alleviation of histopathological changes in kidney tissues. Conclusion: The current findings support that co-administration of geraniol with methotrexate may attenuate methotrexate-induced acute kidney injury.

## 1. Introduction

Acute kidney injury (AKI) is associated with the development and progression of chronic kidney diseases (CKD) as well as increased mortality and morbidly [1,2]. Drug-induced nephrotoxicity is usually related to the molecular characteristics and metabolism of drugs as well as their ability to crystallize and precipitate within the tubular lumen [3]. Methotrexate (MTX), a dihydrofolate reductase inhibitor, is a disease-modifying drug used for the treatment of neoplastic diseases, psoriasis, rheumatoid arthritis and lupus erythematosus [4,5]. Because 90% of MTX is largely cleared via the kidneys, severe renal toxicity is one of the factors limiting the use of MTX clinically [2]. In addition, MTX and its 7-hydroxy metabolite have a tendency to crystallize and precipitate in the renal tubules in low urinary pH and sluggish urine flow rates [4,5]. Although crystallization in the renal tubules is the most commonly described mechanism of MTX nephrotoxicity, other mechanisms have been described. MTX induces mitochondrial dysfunction [5], neutrophil infiltration and activates neutrophils [6] and oxidative stress [5,6]. Furthermore, MTX has a direct action on the mesangial cells, tubular epithelial cells and afferent capillary constriction [7,8].

Previous studies proved that free radicals and reactive oxygen species (ROS) induce disturbance of the oxidant–antioxidant balance leading to the activation of several redox-sensitive pathways including nuclear-factor-erythroid-2-related factor 2 (Nrf-2)-Kelch ECH associating protein 1 (Keap-1) pathway, which has a vital role in protection against stress insults caused by ROS [9]. Nrf2 is a basic leucine zipper protein that regulates the expression of protective and antioxidant genes, including NAD (P)H quinone dehydrogenase 1 (NQO-1) and heme oxygenase 1 (HO-1) [10,11]. Under unstressed conditions, Nrf2 is quickly degraded by a cluster of proteins in the cytoplasm. The substrate adaptor protein Keap-1 facilitates the ubiquitination of Nrf2 [12]. By disrupting critical cysteine residues in Keap-1, oxidative stress reduces the ubiquitination of Nrf2, which translocates into the nucleus and activates the transcription of genes that protect against oxidative damage and inflammation [10]. Nrf2 signaling activation has been implicated in ameliorating drug-induced oxidative stress and organ damages [11,13,14]. On the contrary, Nrf2 deficiency was associated with a greater severity of renal damage in a mouse model of ischemic and nephrotoxic AKI [15].

Another pathway is mitogen-activated protein kinases (MAPKs), which encompass a large number of serine/threonine kinases involved in regulating a wide array of cellular processes including proliferation, differentiation, stress adaptation and apoptosis [16]. The p38 MAPK pathway has a vital role in the activation and induction of NF-κB, and it plays a regulatory function of inflammation in various diseases [9] making the MAPK/NF-κB signaling pathway a strategic organizer of the inflammatory processes. MAPK activation was reported in MTX-induced nephrotoxicity [9,17].

Numerous studies have demonstrated the role of oxidative stress and inflammation in MTX toxicity [1,2,4,5,6,7]. Hence, agents or compounds, especially from natural origin, that possess antioxidant/anti-inflammatory may be considered as a candidate to mitigate MTX-induced AKI through both Nrf-2/Keap-1 and p38MAPK/NF-κB pathways, which have become attractive signaling targets for drug activities.

Medicinal plants and their bioactive constituents have been well acknowledged in counteracting drug-induced toxicity [18]. Geraniol (Ger) is an acyclic monoterpene alcohol that is the main constituent of the essential oils of rose, lavender, ginger, lemon and orange [12]. Ger has been reported to have multiple biological and pharmacological effects, such as anti-inflammatory [18], antioxidant [19], anti-ulcer [20], anti-cancer [21], anti-depressant [22], neuroprotective properties [23] and ameliorating diabetic nephropathy [24].

To our knowledge, there is no report regarding the protective effects of Ger administration on MTX-induced AKI in rats. Therefore, the current study was designed to determine the possible protective effects of Ger in MTX-induced AKI. Furthermore, Nrf-2/Keap-1 and p38MAPK/NFκB pathways were proposed and explored as potential signaling targets for the natural compound.

## 2. Materials and Methods

### 2.1. Animals and Ethical Statement

Male Wister rats (weight: 200–230 g) were obtained from the animal house facility, King Saud University, Riyadh. The animals were kept at standard laboratory conditions with free access to pellet diet and tap water, ad libitum, throughout the whole experiment. All animal investigational protocols and practices were applied agreeing with the Ethical Conduct for Use of Animals in Research Guidelines in King Faisal University and following Animal Research Ethics Committee permission at the University.

### 2.2. Experimental Design and Treatments

Wister male rats were divided into five groups (*n* = 6): The control group received 0.5% carboxymethyl cellulose (CMC) in saline for 19 days via oral gavage and a single intraperitoneal (IP) injection of physiological saline at day 16. The Ger group received Ger (100 mg/kg/day) dissolved in 0.5% CMC in saline for 19 days and a single IP injection of physiological saline at day 16. The MTX group received 0.5% CMC in saline for 19 days and a single IP injection of MTX (20 mg/kg; Shanxi PUDE Pharmaceutical Company, Datong, China) at day 16. The MTX + Ger (100 mg/kg) group received Ger (100 mg/kg/day) dissolved in 0.5% CMC in saline for 19 days and a single IP injection of MTX (20 mg/kg) at day 16. The MTX + Ger (200 mg/kg) group received Ger (200 mg/kg/day) dissolved in 0.5% CMC in saline for 19 days and a single IP injection of MTX (20 mg/kg) at day 16. An MTX-induced AKI model was established as reported previously [1,25], and the doses of Ger were selected based on previous studies that showed the in vivo renoprotective effect on diabetic animals [24].

### 2.3. Serum and Tissue Homogenate Biochemical Markers

After 24 h from the final administration (day 19), the rats were anaesthetized with ketamine, and blood samples were collected. For serum separation, blood was collected and centrifuged at 4000× *g* for 10 min at 4 °C and stored at −80 °C until analysis. Rats were sacrificed by cervical dislocation, then, kidneys were surgically isolated, washed in ice-cooled saline, dried and weighed. Renal tissue homogenates were prepared (10% *w*/*v*) in ice-cooled phosphate buffered saline (PBS) using homogenizer and centrifuged for 10 min at 4000× *g* at 4 °C, where the obtained supernatant was used for estimation of renal biochemical parameters and molecular biomarkers. The other kidney was used for histopathological examination.

### 2.4. Determination of Kidney Index

Body weight of the animals was measured at the initial (initial body weight) and the final (final body weight) stages of the experimental period. Bodyweight (gm) was measured, and relative kidney weight (kidney index) was calculated according to:

Kidney index = (kidney weight/final body weight) × 100

### 2.5. Determination of Renal Function

Renal function parameters, including creatinine, BUN, uric acid and albumin, were colorimetrically determined in serum using specified kits according to the manufacturer’s protocols using spectrophotometer (LEICA UNISTAT^®^; Leica Inc., Allendale, NJ, USA). In addition, kidney injury molecule-1 (Kim-1) was measured in kidney homogenate using ELISA kit.

### 2.6. Determination of Oxidative Stress and Antioxidant Markers

Malondialdehyde (MDA) [26] and NO, assayed using Griess reagent [27], reduced glutathione (GSH; Abcam Co., Eugene, OR, USA; Cat. No. ab102530), superoxide dismutase (Abcam Co.; Cat. No. ab65354), catalase (CAT; Abcam Co., Eugene, OR, USA; Cat. No. ab83464) and glutathione peroxidase (GSHPx; Abcam Co., Eugene, OR, USA; Cat. No. ab102530) were measured in the kidney homogenate samples using ELISA kit according to the manufacturer’s protocols.

### 2.7. Histological Examination

Kidney samples were fixed in 10% neutral buffered formalin for 48 h, then processed via routine histological (dehydration, clearing and paraffin embedding). Sections of 5 μm were cut, stained with hematoxylin and eosin (H&E) and examined using a light microscope. Renal injury was evaluated in the renal sections by a blinded observer unaware of the experimental groups. The severity of tubular injury was considered as a percent of tubules of the section showing a given tubular alteration (tubular dilatation/flattening, loss of brush border, vacuolated cells, cellular detachment, focal necrosis, intraluminal nuclei and debris) and was graded as follows: 0, less than 5%; 1, 5–33%; 2, 34–66% and 3, over 66%, as previously described [28].

### 2.8. Real-Time PCR

Real-time PCR was performed according to the technique described elsewhere [25]. Briefly, the Trizol reagent kit (Invitrogen, Waltham, MA, USA) and reverse transcription polymerase chain reaction (RT-PCR) kit (TaKaRa, (Shiga, Japan), Cat. No. RR037A) were used to cleanse total RNA and inverse transcription reaction, respectively. In total, 20 μL of the reaction volume was mixed with 1 μL total RNA (1 μg/μL), incubated at 42 °C for 15 min, followed by 95 °C for 2 min, and the generated cDNA was stored at −20 °C. In total, 50 μL of PCR reaction mixture enclosed ×50 ROX Reference Dye (1 μL), sense and antisense primers (1 μL each, primers are mentioned in Table 1), ×2 SYBR Green PCR Master Mix (25 μL), cDNA template (4 μL) and sterilized distilled H2O (18 μL). The PCR reaction condition incorporated pre-denaturing at 95 °C for 10 s, then 40 cycles of 95 °C/5 s and 60 °C/30 s and 72 °C/1 min. Quantification analyses were completed via Opticon-2 Real-Time PCR reactor (MJ Research, Reno, NV, USA). Step PE Applied Biosystems (Perkin Elmer, Waltham, MA, USA) software was used to analyze real-time PCR results. Expression of the target gene was measured and correlated to the reference gene (β-actin).

### 2.9. Western Blot

Western blot was performed according to the method described previously [29] to assess Keap-1/Nrf-2/HO-1 and p38 MAPK/NF-κB pathways’ protein expression. Briefly, renal tissue samples were mixed with RIPA buffer containing protease inhibitor; the extracted protein was measured using a Nano Drop Lite spectrophotometer (Thermo Fisher Scientific, Waltham, MA, USA). Thereafter, 50 μg of the total extracted protein was separated via SDS-PAGE and blotted onto PVDF membranes. PVDF membranes were blocked by incubation in TBS enclosing 3% bovine serum albumin and 0.1% Tween 20 for one hour at room temperature. PVDF membranes were washed (TBS containing 0.1% Tween 20) and incubated first with a 1:1000 dilution of the primary antibodies (Keap-1, Nrf2, HO-1, P38 MAPK and NF-κB) for two hours and, then, with a 1:5000 dilution of the secondary antibody at room temperature. Keap-1 (sc-514914), Nrf2 (sc-722), HO-1 (sc-136960), P38 MAPK (sc-7973), NF-κB p65 (sc-8008), reference gene (β-actin; SC-130656) and goat anti-rabbit immunoglobulin (Ig) G-horseradish peroxidase (HRP) (sc-2030) antibodies were purchased from Santa Cruz Biotechnology, Inc. (Dallas, TX, USA). The chemiluminescence produced was detected with the C-DiGit chemiluminescence scanner (LI-COR, Lincoln, NE, USA), and the band intensity was analyzed using the scanner software.

### 2.10. Determination of Inflammatory Mediators

Inflammation markers including TNF-α (Abcam Co., Eugene, OR, USA; Cat. No. ab46070), IL-1β (Abcam Co., Eugene, OR, USA; Cat. No. ab100768), IL-6 (Abcam Co., Eugene, OR, USA; Cat. No. ab100772) and IL-10 (Abcam Co., Eugene, OR, USA; Cat. No. ab133112) were measured in kidney homogenate via ELISA kits following the manufacturers’ instructions using a microplate reader SpectraMax i3X (Molecular devices, San Jose, CA, USA).

### 2.11. Determination of Apoptotic Signaling Markers

Cleaved caspase-3 and caspase-9 were assessed in kidney homogenate using the specified ELISA kits following the manufacturers’ instructions using a microplate reader SpectraMax i3X (Molecular devices). Bcl 2 and Bax mRNA were measured using real-time PCR, as mentioned earlier.

### 2.12. Statistical Analysis

Data are presented as mean ± SD. For multiple comparisons, one-way ANOVA followed by Tukey–Kramer as a post hoc test was performed. The 0.05 level of probability was used as the significance level. All statistical analyses were performed using Graph Pad software (version 5, San Diego, CA, USA).

## 3. Results

### 3.1. Ger Declined Kidney Index in MTX-Induced AKI in Rats

There is no significant difference between the animals groups regarding the initial body weight. The MTX group showed a significant decrease in final body weight and an increase in kidney index in comparison with the normal control. Pre-treatment of the MTX administered rats with Ger showed a significant increase in final body weight with a decrease in kidney index when compared to MTX-only-administered animals (Table 2). However, the change in kidney index was more related to the change in body weight rather than the change in kidney weight.

### 3.2. Ger Enhanced Renal Histopathological Alterations in MTX-Induced AKI in Rats

**Figure 1:**
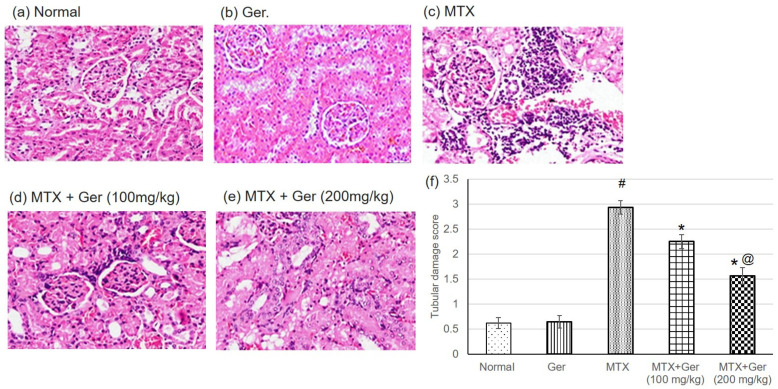
Effects of treatment with Ger (100 and 200 mg/kg) for 19 days on the histopathological investigation using H&E staining demonstrating normal glomeruli and renal tubules in (**a**) normal and (**b**) Ger groups, congestion of interstitial blood vessel and glomerular capillary tuft associated with intense focal renal interstitial proliferating mononuclear cell infiltration in MTX group (**c**), focal necrosis of epithelial lining associated with uniformly arranged regenerative renal tubules in Ger-administered groups (**d**) and (**e**) (H&E, X400) and (**f**) tubular damage score, which varies from 0 for completely normal histology to 3 for maximal and widespread injury. All values are expressed as mean ± SD. #, statistically significant from normal group; *, statistically significant from MTX-only group; @, statistically significant from MTX + Ger 100 mg/kg group (*p* < 0.05) using one-way ANOVA followed by Tukey’s post hoc analysis.

H&E staining indicated severe histological kidney injuries including congestion of interstitial blood vessel and glomerular capillary tuft associated with intense focal renal interstitial proliferating and mononuclear cell infiltration in MTX group (Figure 1c). Geraniol administration prior to MTX administration showed focal necrosis of epithelial lining associated with uniformly arranged regenerative renal tubules (Figure 1d,e).

### 3.3. Ger Averted MTX-Induced Kidney Injury in Rats

**Figure 2:**
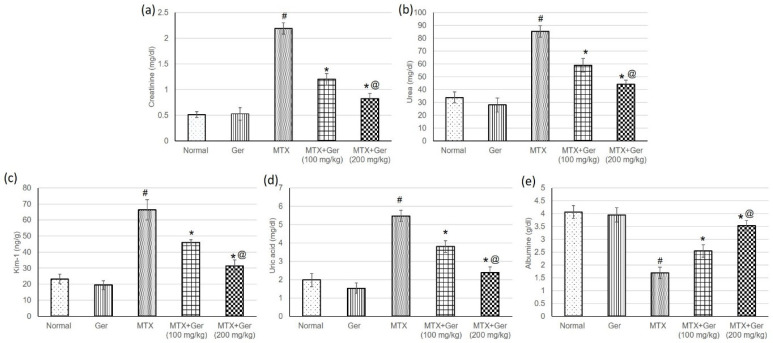
Effects of treatment with Ger (100 and 200 mg/kg) for 19 days on the kidney function including (**a**) creatinine, (**b**) urea, (**c**) Kim-1, (**d**) uric acid and (**e**) albumin in MTX-induced AKI in rats (*n* = 6). All values are expressed as mean ± SD. #, statistically significant from normal group; *, statistically significant from MTX-only group; @, statistically significant from MTX + Ger 100 mg/kg group (*p* < 0.05) using one-way ANOVA followed by Tukey’s post hoc analysis.

To assess the protective effect of Ger on MTX-induced AKI, we evaluated serum levels of creatinine, urea, uric acid, albumin and Kim-1. The transmembrane protein Kim-1 is upregulated in response to proximal convoluted tubules (PCT) injury to suppress apoptosis and promote tubular re-epithelization. Therefore, Kim-1 is a specific and sensitive biomarker of nephrotoxicity [29]. MTX caused a significant intensification in creatinine (Figure 2a), urea (Figure 2b), Kim-1 (Figure 2c) and uric acid (Figure 2d) levels, while serum albumin level (Figure 1e) was significantly decreased as compared to the normal group. Pre-treatment of the MTX-administered rats with Ger significantly prevented MTX-induced changes in serum creatinine, urea, uric acid, albumin and Kim-1as compared to MTX-only-treated rats (Figure 2).

### 3.4. Ger Regulated Renal Keap1/Nrf2/HO-1 Pathway in MTX-Induced AKI

**Figure 3:**
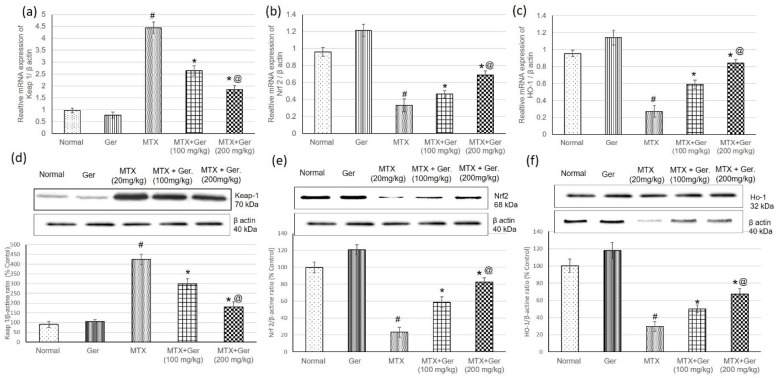
Effects of treatment with Ger (100 and 200 mg/kg) for 19 days on the Keap-1/Nrf2/HO-1 signaling pathway including mRNA expression on (**a**) Keap-1, (**b**) Nrf2 and (**c**) HO-1 levels and on protein expression of (**d**) Keap-1, (**e**) Nrf2 and (**f**) HO-1 levels in MTX-induced AKI in rats (*n* = 6). All values are expressed as mean ± SD. #, statistically significant from normal group; *, statistically significant from MTX-only group; @, statistically significant from MTX + Ger 100 mg/kg group (*p* < 0.05) using one-way ANOVA followed by Tukey’s post hoc analysis.

To study the effect of Ger on Keap-1/Nrf2/HO-1 in the kidney of MTX-induced rats, we determined gene (mRNA) and protein expression levels of Keap1, Nrf2 and HO-1 using qRT-PCR and Western blot techniques, respectively. Assessment of Keap1 levels in renal tissues revealed a significant increase in its levels; however, Nrf2 and HO-1 levels were considerably decreased in rats administered MTX only relative to the normal group (Figure 3). On the other hand, pre-treatment of the MTX-administered rats with Ger reduced Keap1 levels and significantly escalated renal Nrf2 and HO-1 (Figure 3) when compared to the MTX group.

### 3.5. Ger Enhanced Antioxidants Activities in MTX-Induced AKI

Malondialdehyde (MDA) is one of the compounds formed during lipid peroxidation, which has been accepted as a valuable indicator of lipid peroxidation and oxidative stress [30]. Animals administered MTX only showed significant reduction in GSH, SOD, CAT and GSHPx together with an increase in MDA and NO when compared with the control group (Table 3, while pre-treatment of the MTX-administered rats with Ger in both 100 mg/kg and 200 mg/kg doses ameliorated GSH, SOD, CAT and GSHPx as well as reduced MDA and NO when compared to MTX group of animals.

### 3.6. Ger Diminished Inflammatory Response via P38 MAPK/NF-κB Pathway in MTX-Induced AKI

**Figure 4:**
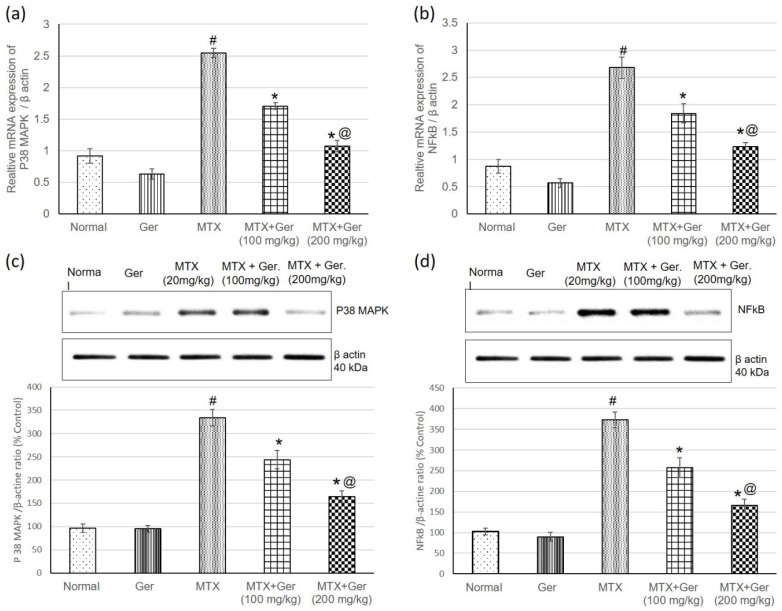
Effects of treatment with Ger (100 and 200 mg/kg) for 19 days on the P38 MAPK/NF-κB signaling pathway: mRNA expression on (**a**) P38 MAPK and (**b**) NF-κB levels and on protein expression of (**c**) P38 MAPK and (**d**) NF-κB levels in MTX-induced AKI in rats (*n* = 6). All values are expressed as mean ± SD. #, statistically significant from normal group; *, statistically significant from MTX-only group; @, statistically significant from MTX + Ger 100 mg/kg group (*p* < 0.05) using one-way ANOVA followed by Tukey’s post hoc analysis.

MTX injection significantly increased gene (mRNA) and protein expression levels of renal P38 MAPK and NF-κB as compared to normal control group. On the other hand, geraniol administration prior to MTX administration showed a significant decrease in P38 MAPK and NF-κB expression in comparison with MTX-only-administered animals (Figure 4), indicating the anti-inflammatory potential of Ger, which could be one of the underlying mechanisms in MTX-induced nephrotoxicity treatment.

### 3.7. Ger Diminished Inflammatory Mediators in MTX-Induced AKI

**Figure 5:**
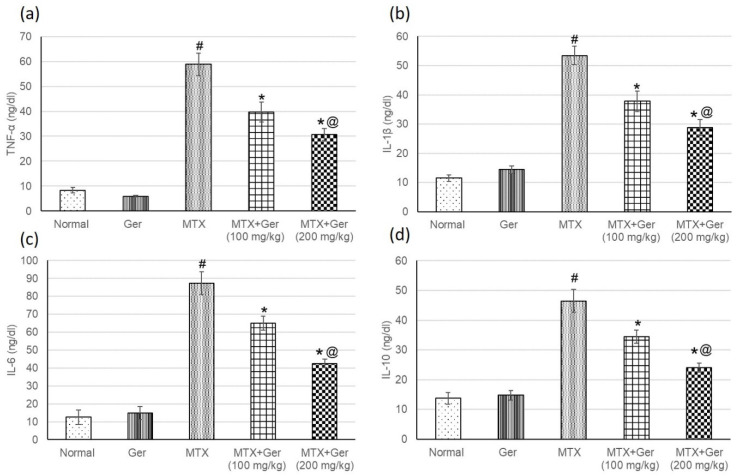
Effects of treatment with Ger (100 and 200 mg/kg) for 19 days on the inflammatory mediators including (**a**) TNF-α, (**b**) IL-1β, (**c**) IL-6 and (**d**) IL-10 in MTX-induced AKI in rats (*n* = 6). All values are expressed as mean ± SD. #, statistically significant from normal group; *, statistically significant from MTX-only group; @, statistically significant from MTX + Ger 100 mg/kg group (*p* < 0.05) using one-way ANOVA followed by Tukey’s post hoc analysis.

NF-κB is a transcription factor that activates the expression of inflammatory mediators. Therefore, after determining the levels of NF-κB, inflammatory mediators including TNF-α, IL-1β, IL-6 and IL-10 were measured to investigate the anti-inflammatory effect of the Ger on MTX-administered animals’ group. MTX-administered animals showed intensification in inflammatory mediators when related with the normal group (Figure 5). The pre-treatment with Ger (100 mg/kg and 200 mg/kg) ameliorated these intensified inflammatory mediators when compared to the MTX group. These outcomes together with P38 MAPK and NF-κB results signified the influential anti-inflammatory effect of Ger.

### 3.8. Ger Mitigated Apoptotic Signaling in MTX-Induced AKI via Regulation of Bax/Bcl2/Caspase-3 and -9

**Figure 6:**
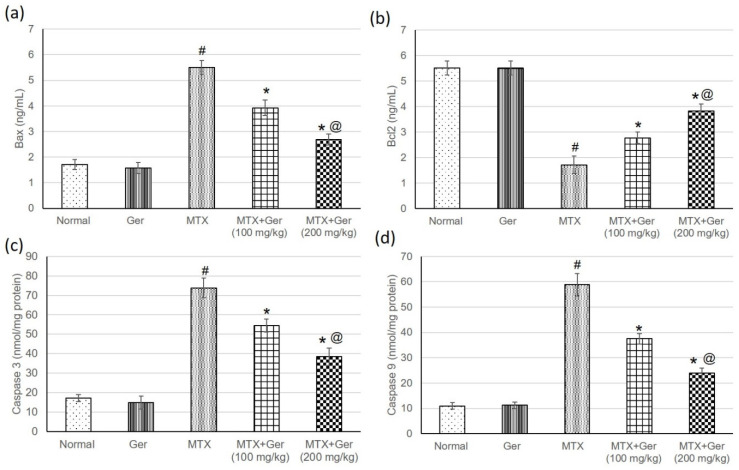
Effects of treatment with Ger (100 and 200 mg/kg) for 19 days on the apoptotic markers including (**a**) Bax, (**b**) Bcl2, (**c**) caspase-3 and (**d**) caspase-9 in MTX-induced AKI in rats (*n* = 6). All values are expressed as mean ± SD. #, statistically significant from normal group; *, statistically significant from MTX-only group; @, statistically significant from MTX + Ger 100 mg/kg group (*p* < 0.05) using one-way ANOVA followed by Tukey’s post hoc analysis.

Rats treated with MTX only exhibited an increase in Bax, caspase-3 and -9 as well as a reduction in Bcl2 in comparison with the normal control. These changes were significantly alleviated by geraniol administration prior to MTX administration through lowering Bax, caspase-3 and -9 and increasing Bcl2 levels with respect to the MTX group (Figure 6).

## 4. Discussion

Despite the accumulating knowledge about MTX-induced nephrotoxicity, effective pharmacotherapies hindering this serious complication are still unavailable. Hence, it is becoming more crucial to find novel therapeutic approaches to prevent and/or treat MTX-induced AKI. Herein, we investigated the protective effect of Ger, a natural monoterpenoid, with encouraging pharmacological actions, against MTX-induced AKI in rats. Our findings demonstrated that Ger effectively prevented renal oxidative injury, inflammation and apoptosis in MTX-administered rats, possibly through augmenting Nrf2/HO-1 and mitigating MAPK/NF-κB signaling pathways.

### 4.1. Ger and the Status of Renal Damage Induced by MTX

In the existing study, MTX administration induced a status of renal damage, as indicated from the significant increase in creatinine, urea, uric acid and Kim-1 levels together with a decrease in albumin concentration. These measurements are normally used as reliable markers of renal damage and indicate the loss of a majority of kidney functions [2,29,31]. These findings are in agreement with several studies, which have demonstrated MTX-induced renal toxicity in both patients and animal models [1,2,25,32]. These biochemical outcomes were further supported by the histopathological examination, which displayed renal blood vessels’ dilatation and congestion, necrotic epithelial cell, infiltrated leucocyte, as well as some atrophied glomeruli with diminuted urinary space. Smeland et al. [33] demonstrated that MTX or its metabolites’ precipitation in the renal tubules resulted in renal clearance obstruction and diminution. Another study performed by Widemann and Adamson [7] has verified that MTX-induced AKI is a result of its crystallization within the renal tubules. On the other hand, Ger administration prior to MTX significantly improved creatinine, urea, uric acid and Kim-1 levels and attenuated albumin and MTX-induced histological alterations in the kidney. In accordance, Ger has shown renoprotective effects in T2DM [24] and in cisplatin-induced toxicity [23].

Accumulating evidence has pointed to the role of oxidative injury and inflammation in provoking apoptosis in MTX-induced AKI [2,3,11]. Therefore, we have proposed two pathways: the suppression of oxidative stress via Nrf2/HO-1 activation and inflammation inhibition via MAPK/NF-κB pathways that may mediate, at least in part, the protective mechanism of Ger against AKI induced by MTX.

### 4.2. Ger and Oxidative Stress Parameters and Pathways

Oxidative stress is a hallmark of MTX-induced nephrotoxicity and one of the main mechanisms through which MTX induces renal tissue damage [1,7]. Therefore, it is currently recommended that MTX usage be combined with antioxidant agents such as folic acid in order to minimize MTX toxicity [25]. MTX has been reported to increase ROS production via suppressing homocysteine remethylation [34], stimulation of neutrophils [6], activation of NADPH oxidase, depletion of NADPH and mitochondrial dysfunction [5]. MTX inhibits enzymes responsible for the production of nicotinamide adenine dinucleotide phosphate (NADPH) in proliferating cells causing glutathione level reduction [3,25]. Excess ROS can also activate nuclear factor-kappa B (NF-κB) and the release of proinflammatory cytokines and trigger mitochondrial dysfunction and apoptosis [11]. Given the role of ROS and oxidative stress in the development of MTX nephrotoxicity, the induction of antioxidant and cytoprotective enzymes is critical.

In the hereby study, MTX administration produced a marked increase in lipid peroxidation and NO levels in the kidney of rats. Moreover, GSH content showed a significant decline in the kidneys of MTX-induced rats. The activity of the antioxidant enzymes SOD, GSHPx and CAT was decreased in the kidneys of the MTX-induced rats. These antioxidant defense enzymes play a key role in protecting against the deleterious effects of ROS. The decreased activity of antioxidant enzymes and depletion of GSH have led to mitochondrial dysfunction, which causes necrosis and impaired renal function [1,3]. Therefore, suppressing ROS generation and the enhancement of cellular antioxidants defenses can mitigate MTX-induced AKI. Ger markedly decreased kidney MDA and NO levels and enhanced both the enzymatic and non-enzymatic antioxidant defenses, signifying the potent free-radical scavenging and antioxidant efficacies of the compound. Accordingly, multiple studies have demonstrated the antioxidant potential of Ger in kidney injury induced by cisplatin [23] and diabetic nephropathy [24]. Furthermore, the antioxidant potential of Ge was revealed in different conditions, for instance, traumatic spinal cord injury (SCI) [35], aluminum chloride-hepatopancreatic toxicity [36], osteoarthritis (OA) [37], acetic acid and helicobacter pylori-induced gastric ulcers [38].

The enhanced efficacy of the antioxidant defense system could be directly connected to the ability of Ger to positively regulate the Keap1/Nrf2/HO-1 signaling pathway. MTX administration produced a significant downregulation of Nrf2 and HO-1 abundance, an effect that was potentially reversed by prior administration of Ger. Nrf2 plays a crucial role in regulating the basal and inducible expressions of several cytoprotective and antioxidant genes, which can counteract oxidative stress [32]. Although exposure to moderate oxidative stress leads to Nrf2 activation, excessive and sustained ROS generation can diminish Nrf2 signaling in the kidney [2,3,11,32] and liver [2,39] of rats challenged with MTX. Thus, the diminished Nrf2/HO-1 pathway is a direct consequence of the sustained ROS generation induced by MTX. Ger administration upregulated renal Nrf2, with the consequent induction of HO-1, CAT and SOD in rats challenged with MTX. Upregulated Nrf2 signaling by Ger resulted in enhanced antioxidants and diminished ROS and oxidative damage. Furthermore, the activation of Nrf2 had a key role in the anti-inflammatory and antiapoptotic effects of Ger. Nrf2 and HO-1 can directly inhibit NF-κB signaling and pro-inflammatory cytokines and activate the anti-inflammatory cytokines, thereby regulating the inflammatory cascade as we will discuss later. Therefore, the observed induction of antioxidant enzymes by Ger could be explained, at least in part, by the functional activation of the Nrf2/HO-1 signaling pathway in the kidneys of MTX-induced rats. The role of Nrf2 in mediating the anti-inflammatory and antiapoptotic efficacies of Ger in MTX-administered rats was supported by previous reports, which demonstrated increased expression of Nrf2 and suppressed inflammation in hepatic ischemia-reperfusion injury [40], diet-induced experimental atherosclerosis [41] and in ovalbumin-induced allergic asthma [18].

### 4.3. Ger and Inflammation Parameters and Pathways

MTX nephrotoxicity has been associated with both inflammation and apoptosis [32]. Inflammatory cytokines activate neutrophils and macrophages, increase ROS and, subsequently, provoke neutrophil infiltration in the kidney [1]. The predominant mechanism by which NF-κB is activated by various stimuli is through the phosphorylation of IkB, an inhibitory protein that under normal conditions binds to NF-κB and sequesters it in the cytoplasm, thereby preventing its access to DNA. The phosphorylation of IkB results in its ubiquitination and degradation, freeing NF-κB to translocate to the nucleus and activate transcription [16]. We proved in this study that MTX significantly intensified the levels of renal P38 MAPK, NF-κB and other inflammatory mediators, while the co-administration of Ger declined all these parameters. The inhibitory effect of Ger on the MAPK signaling pathway was previously reported in other conditions such as cisplatin-induced neurotoxicity [23], cyclophosphamide-induced hepatotoxicity [42], TNBS-induced colitis [43] and osteoarthritis [37]. Ger was considered an effective adjuvant therapy for cisplatin-induced neurotoxicity that acts through inhibiting the consequences of increases in p21, FMO3, MMP9, p38 MAPK, STAT-1 and p53 gene expression, glutamate levels and oxidative stress in brain tissues [23].

### 4.4. Ger and Apoptotic Signaling

In the current study, increased ROS and inflammation were associated with oxidative DNA damage and upregulated Bax and caspases. Bax is a proapoptotic protein that elicits cytochrome c release from the mitochondria and consequent activation of caspases. In addition, excessive mitochondrial ROS production during MTX metabolism can damage the mitochondrial membrane, resulting in the loss of membrane potential and, consequently, the release of cytochrome c, which ultimately culminates in renal apoptosis by activating caspase-3 [1,32]. The kidneys of MTX-induced rats in this study showed increased Bax/Bcl2 ratio, caspases-3 and -9, demonstrating activated apoptotic signaling. Therefore, the activation of MAPK and its subsequent NF-κB stimulation of and release of inflammatory cytokines provoked apoptosis in the kidney of MTX-induced rats. Increased ROS production could be considered as the main culprit behind activation of the MAPK/NF-κB signaling in the kidneys of MTX-induced rats. The inhibition of MTX-mediated ROS generation and proinflammatory cytokines production can protect against apoptosis. Given its dual ability to attenuate oxidative injury and inflammation, geraniol administration prior to MTX prevented apoptotic signaling induced by MTX as shown by the diminished Bax and caspases and upregulation of the antiapoptotic Bcl2. In the same context, previous studies have shown that Ger suppressed apoptosis in LPS-induced acute lung injury [44], hepatic ischemia-reperfusion injury [40], isoproterenol-induced cardiotoxicity [12], traumatic injury of the spinal cord [35], chronic MPTP/probenecid-induced Parkinson’s disease [45] and others.

## 5. Conclusions

In the current investigation, we evaluated the protective effects of Geo MTX-induced nephrotoxicity in rats. We retrieved that geraniol administration prior to MTX protected against kidney injury progression through upregulating the Keap1/Nrf2/HO-1 pathway resulting in attenuating oxidative stress and diminishing MAPK and NF-kB with subsequent inflammation-limiting effects. This dual antioxidant and anti-inflammatory activities of Ger allowed us to counteract the apoptotic effect of MTX on kidney tissues. This study represents an added value to Ger, the edible, safe and medicinally and pharmacologically active monoterpene.

## Figures and Tables

**Table 1:** Primer sequences for all markers used in real-time PCR experiments.

Markers	Primer Sequence (5′ to 3′)	
	Forward Primer	Reverse Primers
Bcl-2	5′-CCGGGAGATCGTGATGAAGT-3′	3′-ATCCCAGCCTCCGTTATCCT-5′
Bax	5′-GTGGTTGCCCTCTTCTACTTTG-3′	3′-CACAAAGATGGTCACTGTCTGC-5′
Nrf2	5′-CATTTGTAGATGACCATGAGTCGC-3′	3′-ATCAGGGGTGGTGAAGACTG-5′
Keap-1	5′-CTTCGGGGAGGAGGAGTTCT-3′	3′-CGTTCAGATCATCGCGGCTG-5′
HO-1	5′-GTGCACATCCGTGCAGAGAA-3′	3′-GTGCACATCCGTGCAGAGAA-5′
P38 MAPK	5′-AGAGTCTCTGTCGACCTGCT-3′	3′-CCTGCTTTCAAAGGACTGGT-5′
NF-κB	5′-TGGGACGACACCTCTACACA-3′	3′-GGAGCTCATCTCATAGTTGTCC-5′
β-actin	5′-TGACAGGATGCAGAAGGAGA-3′	3′-TAGAGCCACCAATCCACACA-5′

**Table 2:** Effects of treatment with geraniol on body weight and kidney index in MTX-induced AKI in rats.

Treated Groups	Body Weight (gm)		Kidney Index
	Initial Body Weight	Final Body Weight	
Normal	190 ± 8.68	275 ± 14.5	0.71 ± 0.04
Geraniol	200 ± 11.46	280 ± 17.6	0.72 ± 0.03
MTX (20 mg/kg)	195.72 ± 19.22	140.62 ± 8.96 ^#^	1.19 ± 0.08 ^#^
MTX + Ger (100 mg/kg)	195.48 ± 21.15	185.00 ± 12.76	0.87 ± 0.10 *
MTX + Ger (200 mg/kg)	210.00 ± 20.87	198.00 ± 13.7	0.85 ± 0.08 *

All values are expressed as mean ± SD. #, statistically significant from normal group; *, statistically significant from MTX-only group, (*p* < 0.05) using one-way ANOVA followed by Tukey’s post hoc analysis.

**Table 3:** Effects of treatment with geraniol on antioxidants activities in MTX-induced AKI in rats.

	Normal	Ger	MTX (20 mg/kg)	MTX + Ger (100 mg/kg)	MTX + Ger (200 mg/kg)
MDA (nmol/100 mg)	22.60 ± 3.65	25.34 ± 5.32	92.83 ± 8.37 ^#^	34.96 ± 5.04 *	30.64 ± 4.15 *
NO (nmol/100 mg)	21.39 ± 0.30	20.76 ± 3.59	79.24 ± 0.38 ^#^	52.46 ± 0.22 *	32.01 ± 0.18 *^,@^
Reduced GSH (nmol/100 mg)	69.56 ± 8.48	67.12 ± 13.76	22.91 ± 2.12 ^#^	32.27 ± 3.51 *	48.97 ± 6.51 *^,@^
GSHPx (U/g protein)	0.060 ± 0.01	0.062 ± 0.0093	0.045 ± 0.02	0.051 ± 0.01 *	0.055 ± 0.02 *^,@^
SOD (U/g protein)	292.92 ± 22.43	300.32 ± 24.61	156.28 ± 0.69 ^#^	205.33 ± 22.4 *	253.87 ± 11.31 *^,@^
CAT (nmol/g tissue)	76.13 ± 5.34	82.23 ± 15.28	22.70 ± 0.66 ^#^	34.80 ± 5.16 *	48.75 ± 4.38 *^,@^

All values are expressed as mean ± SD, (*n* = 6). #, statistically significant from normal group; *, statistically significant from MTX-only group; @, statistically significant from MTX + Ger 100 mg/kg group (*p* < 0.05) using one-way ANOVA followed by Tukey’s post hoc analysis.

## Data Availability

Not applicable.
